# Maintenance intravenous iron in hemodialysis patients to minimize erythropoietin doses: a double-blinded, randomized controlled trial (the MAINTAIN IRON trial)

**DOI:** 10.1038/s41598-023-28440-3

**Published:** 2023-01-23

**Authors:** Suthiya Anumas, Aphichat Chatkrailert, Pichaya Tantiyavarong

**Affiliations:** 1 Division of Nephrology, Department of Medicine, Faculty of Medicine, Thammasat University, Pathumthani, 12120 Thailand; 2 Chulabhorn International College of Medicine, Thammasat University, Pathumthani, 12120 Thailand; 3 Department of Clinical Epidemiology, Faculty of Medicine, Thammasat University, Pathumthani, 12120 Thailand

**Keywords:** Haemodialysis, Clinical trial design

## Abstract

In patients on chronic hemodialysis, there is no standard protocol for maintenance iron supplementation. This study aimed to compare two fixed-dose intravenous (IV) iron protocols to reduce erythropoiesis-stimulating agents (ESA). We conducted a double-blinded, randomized controlled study on hemodialysis patients having ferritin levels between 200 and 700 ng/dl and transferrin saturation values between 20 and 40%. Patients were assigned to receive either 100 or 200 mg of IV iron each month. ESA was adjusted every month to keep Hb between 10 and 12 g/dl. ESA dose at 12 months was the primary outcome. The secondary outcomes were all-cause mortality, cardiovascular events, absolute iron deficiency anemia (IDA), blood transfusion, adverse events, and iron withholding rate. Of the 79 eligible patients, 40 received 100 mg of IV iron, while 39 received 200 mg. At month 12, the mean monthly ESA dose in the 100-mg IV iron group was 35,706 ± 21,637 IU, compared to 26,382 ± 14,983 IU in the 200-mg group (*P* = 0.03). IDA was found in twelve patients (30%) in the 100-mg group and four patients (10.5%) in the 200-mg group (*P* = 0.05). In each group, three patients died (*P* = 0.9). Hospitalization, venous access thrombosis, and infection rates were similar in both groups. The withholding rate of IV iron was higher in 200-mg group (25% vs*.* 64.1%), but the protocol compliance was found more in 100-mg group (50% vs. 28.2%) (*P* = 0.001). In conclusion, monthly 200-mg IV iron infusions significantly reduce ESA doses but have a higher withholding rate. (Funded by the Kidney Foundation of Thailand and the Research Group in Nephrology and Renal Replacement Therapy from the Faculty of Medicine, Thammasat University).

Thai Clinical Trials Registry number, TCTR20190707001.

## Introduction

Iron deficiency anemia (IDA) remains a major problem in chronic hemodialysis patients^1–3^. Poorer dietary intake, impaired gut absorption, iron depletion from frequent blood samplings, occult gastrointestinal losses, and blood retention in the hemodialysis circuit, all create a negative iron balance^4–6^. Estimated iron loss was approximately 1–3 g per year, equivalent to 83–250 mg per month^7^. Inadequate iron replacement may lead to absolute iron deficiency, commonly defined as when transferrin saturation (TSAT) < 20% and ferritin < 200 ng/dl^1,4,5^.

The PIVOTAL trial^8^ showed decreases in composite cardiovascular endpoints, erythropoiesis-stimulating agents (ESA) doses, and blood transfusions due to the scheduling of regular intravenous (IV) iron replacements in the proactive group. This was versus the reactive group, in which repletion only occurred if the iron deficiency was documented. The trial confirmed the benefits of maintenance iron therapy in hemodialysis patients; however, the iron doses used did not follow the initial study protocol of 400 mg/month. Iron therapy was withheld for safety when TSAT > 40% and/or ferritin > 700 µg/dl. The median actual iron dose was 264 mg/month instead.

Our aim was to find appropriate doses of maintenance iron therapy. Therefore, we conducted a randomized controlled trial to compare the efficacy of 100 mg IV iron with 200 mg IV iron per month regimens to minimize monthly ESA doses. We also explored the incidence of death, absolute IDA, blood transfusion, quality of life, changes in hemoglobin (Hb), ferritin levels, TSAT, and other adverse effects.

## Methods

### Trial design

A single-center, double-blinded, post-hoc superiority, randomized controlled trial was conducted at Thammasat University Hospital from July 2019 to February 2021. The trial protocol was approved by the Human Research Ethics Committee of Thammasat University No 1 (Faculty of Medicine): MTU-EC-OO-4-055/62 and followed the principles of the Declaration of Helsinki and the International Conference on Harmonization Good Clinical Practice guidelines. Written informed consent was obtained from all eligible participants, and the protocol was registered in the Thai Clinical Trials Registry with study number TCTR20190707001 (Date of registration 07/07/2019). This trial was funded by the Kidney Foundation of Thailand and the Research Group in Nephrology and Renal Replacement Therapy, Faculty of Medicine, Thammasat University. Study data were collected and managed using REDCap electronic data capture tools hosted at the Faculty of Medicine, Thammasat University, Thailand^9,10^.

Random allocation was done by computer-generated permuted blocks of varying sizes (blocks of 2 and 4). Randomization was stratified by the level of baseline Hb: < 10, 10–12 and > 12 g/dl. The investigators generated a random allocation sequence, enrolled participants, and assigned treatments. The patients were randomly assigned in a 1:1 to receive either a single, monthly dose of 100 mg or 200 mg IV iron sucrose. Since iron sucrose was mixed with 100-ml saline bags and dripped in 30 min during hemodialysis sessions, patients and care physicians cannot distinguish between both regimens (double-blinded).

After randomization, trial visits were conducted every month up until 12 months. Hb levels were measured monthly; if levels were below or above target levels of 10–12 g/dl, ESA doses were adjusted to either increase or decrease ~ 25% of previous amounts. A blood transfusion would be given if Hb levels were extremely low (usually < 8 g/dl) or anemic symptoms were presented. Ferritin levels and TSAT were measured every three months and maintained in the safety ranges: ferritin 200–700 ng/dl and TSAT 20–40%. Suppose ferritin levels > 700 ng/dl or TSAT > 40%, IV iron was withheld for one month or longer until levels declined below those safety margins. Iron therapy was temporarily withheld if patients had a systemic or severe infection. In patients with absolute IDA, defined as ferritin levels < 200 ng/dl or TSAT < 20%, the treatment was unblinded, and IV iron sucrose was prescribed to be 100 mg weekly for a total of 10 doses (rescue regimen). After this, these patients would receive 200 mg IV iron monthly until the end of the study. Quality of life, measured by the EQ-5D questionnaire, was evaluated every six months. The incidence of death, hospitalizations, and adverse events were recorded throughout the study.

### Participants

Adults ≥ 18 years old with end-stage renal disease, undergoing chronic hemodialysis > 90 days were enrolled in this study. Other eligibility criteria were those with a ferritin level of 200–700 ng/dl, TSAT of 20–40%, Hb > 9 g/dl, and received ESA regularly. Exclusion criteria were life expectancy < 6 months, those planning to receive a kidney transplant or switch to peritoneal dialysis in the next 6 months, active infection, active malignancy, known HIV or hepatitis B or C infection, chronic liver disease, advanced heart failure (NYHA IV), pregnancy or breastfeeding, hematologic malignancy, and previous hypersensitivity reaction to IV iron sucrose.

### Outcomes

The primary efficacy endpoint was to compare mean monthly doses of ESA at month 12 between the two groups. Secondary efficacy endpoints were the incidence of death, myocardial infarction, stroke, IDA, blood transfusion, quality of life, and changes in Hb, ferritin levels, TSAT, and ESA doses.

Safety parameters were evaluated throughout the study, including the incidence of vascular thrombosis, hospitalization, and iron withholding rate.

### Statistical analyses

According to the PIVOTAL trial^8^, the median monthly doses of ESA in the proactive and reactive regimens were 29,757 IU [interquartile range (IQR): 18,673–48,833] and 38,805 IU (IQR: 24,377–60,620), with the median monthly dose of IV iron sucrose of 264 mg and 145 mg, respectively. Thus, we assumed a monthly ESA dose of 30,000 IU for the 200-mg IV iron group and 37,500 IU for the 100-mg IV iron group (25% higher). The sample size was estimated from the 2-sample mean test (Stata v.14.2), which provided our trial with 90% power and alpha 0.05 (one-sided). Allowing for a 10% dropout rate, we needed to enroll at least 72 patients. In the initial study protocol, we aimed to test whether 100-mg IV iron was not inferior to 200-mg. However, after discussion during analyses, a superiority rationale was more accurate because there was no current standard protocol for the maintenance IV iron. Therefore, all analyses were performed in a superiority manner.

Continuous variables were expressed as mean ± standard deviation (SD) or median and IQR and compared using unpaired *t*-test or Mann–Whitney U test, as appropriate. Categorical variables were expressed as frequency and percentages and compared using a Chi-square test or Fisher’s exact test. A linear random-intercept model was used to compare ESA dose at baseline, month 6 and 12, between two treatment regimens. Most of the analyses used an intention-to-treat approach. Though, in patients who died or were transferred to another hemodialysis unit, we did not necessarily know ESA dose, Hb, ferritin levels, and TSAT; therefore, we analyzed only patients who got all these data, which we called the modified intention-to-treat population.

We presented time to withholding IV iron and time to iron rescue therapy between groups using the Kaplan–Meier curve and compared it with Cox proportional hazard models. Mean cumulative doses of IV iron between groups were compared with linear random-intercept model. The patterns of protocol compliance were presented and compared with exact probability test. Moreover, we performed post-hoc subgroup analyses to assess whether sub-populations (defined using median values of baseline Hb, ferritin, and TSAT) may have affected the IV iron therapy withholding rate. And we also test whether certain clinical features may influence the difference in ESA doses.

All *P* values were 2-sided, and *P* < 0.05 was considered statistically significant. All statistical analyses were performed using Stata v.16.0 (StataCorp).

## Results

### Participant characteristics

**Figure 1:**
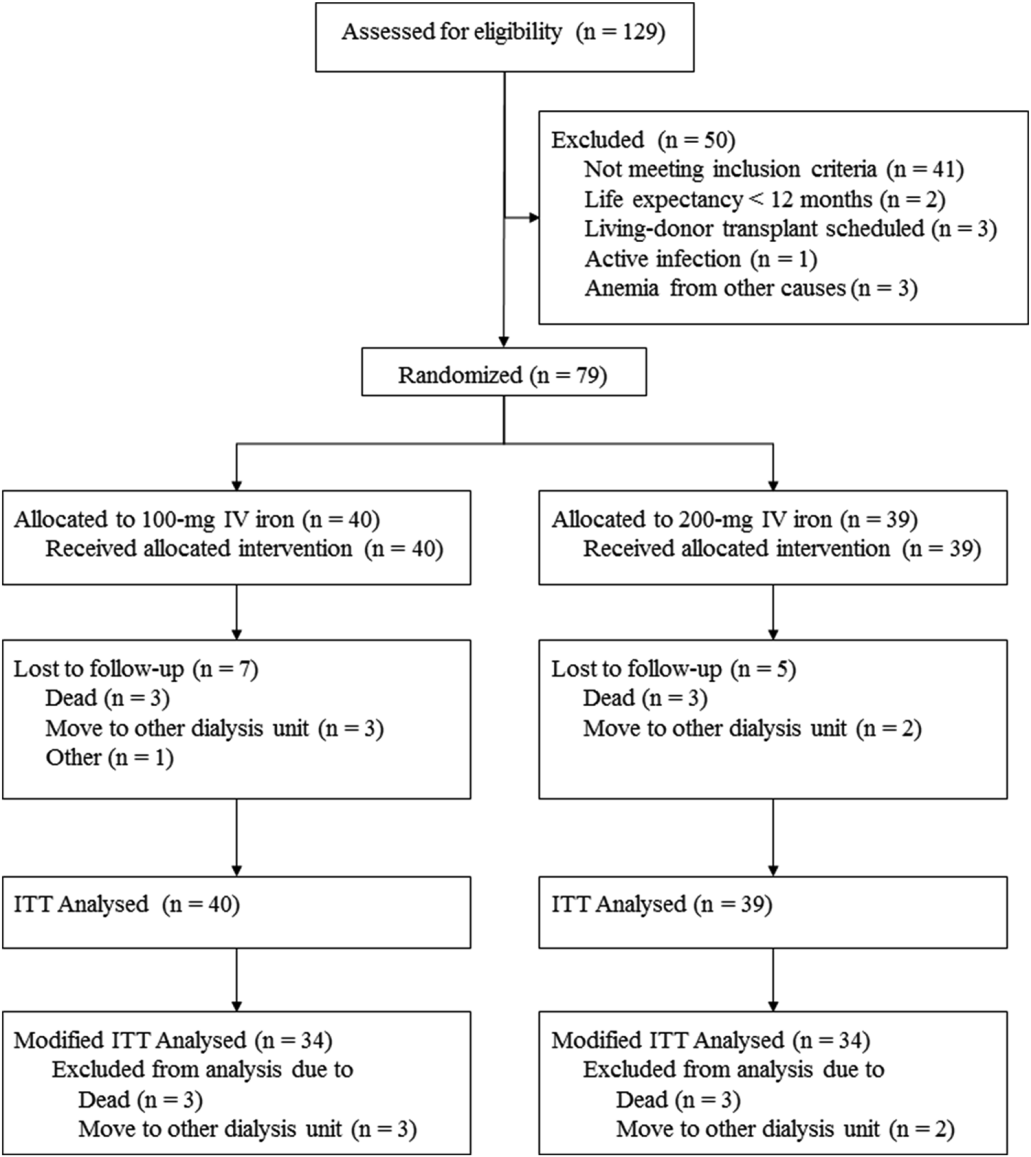
Trial profiles (CONSORT diagram).

Of the 129 patients screened between July 2019 to February 2020 for entry into the trial, 50 did not meet the criteria for randomization (Fig. 1). Of 79 eligible patients, 40 were randomly assigned to the 100-mg IV iron group and 39 to the 200-mg IV iron group. Each patient was followed every month until 12 months. Patients who died and changed the hemodialysis unit during follow-up were excluded from the analysis of the primary endpoint (modified intention-to-treat analysis): 6 in the 100-mg IV iron group and 5 in the 200-mg IV iron group.

**Figure 2:**
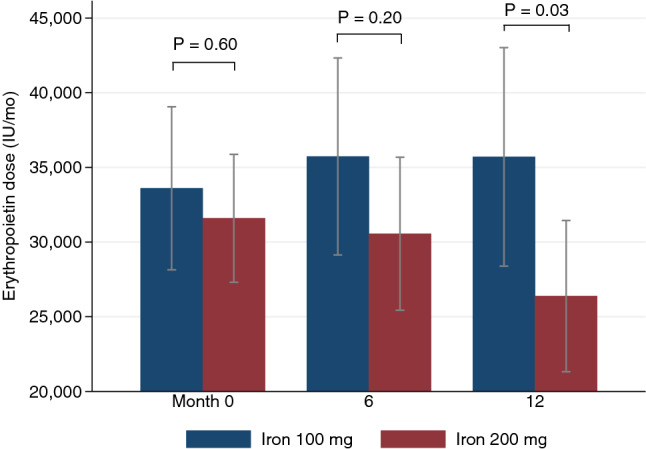
Mean monthly dosages of erythropoietin at month 0 (baseline), month 6, and month 12 (primary endpoint) between 100 and 200-mg intravenous iron groups. The bars indicate 95% confidence intervals. The mean monthly erythropoietin doses at month 12 were 35,706 ± 21,637 IU in 100-mg intravenous iron group and 26,382 ± 14,983 IU in 200-mg group (*P* = 0.03).

The average age was 70.8 ± 11.5 years; hemodialysis duration had a median of 47 months [IQR, 16.9–77.4 months]; and history of coronary artery disease and stroke was 27.9% and 10.1%. Patient baseline characteristics were generally well-balanced in both cohorts (Table 1). Those in the 100-mg IV iron group were older, more women, and had more cardiovascular diseases (atrial fibrillation, peripheral vascular disease, coronary artery disease, and cerebrovascular disease), but there were no statistically significant differences overall. The parathyroid level, C-reactive protein, and use of angiotensin-converting-enzyme inhibitors or angiotensin-receptor blockers were similar in both groups. The mean level of Hb was in a target range of 10–12 g/dl in both cohorts, and the average monthly iron dose was 116.7 ± 73.7 mg in the 100-mg IV iron group and 174.8 ± 74.4 mg in the 200-mg IV iron group (Fig. 2).

**Table 1:** Baseline characteristics of patients.

Characteristics^a^	Iron 100 mg	Iron 200 mg
	(n = 40)	(n = 39)
Age–years	72.6 ± 12.4	68.9 ± 10.4
Male gender—no (%)	14 (35.0)	21 (53.9)
Duration of dialysis^b^—months	53.9 [15.7, 83.2]	42.2 [18.2, 62.4]
Vascular access—no (%)		
Dialysis catheter	15 (37.5)	11 (28.2)
Arteriovenous fistula	22 (55.0)	28 (71.8)
Arteriovenous graft	3 (7.5)	0
Underlying disease—no (%)		
Diabetes	20 (50.0)	21 (53.9)
Hypertension	39 (97.5)	39 (100)
Dyslipidemia	29 (72.5)	28 (71.8)
Atrial fibrillation	6 (15.0)	6 (15.4)
Peripheral vascular disease	3 (7.5)	0
Coronary artery disease	13 (32.5)	9 (23.1)
Cerebrovascular disease	7 (17.5)	1 (2.6)
Smoking status—no (%)		
Currently smoking	0	0
Never smoked	34 (85.0)	25 (64.1)
Former smoker	6 (15.0)	14 (35.9)
Primary kidney disease—no (%)		
Diabetic nephropathy	19 (47.5)	18 (46.2)
Hypertensive nephropathy	13 (32.5)	9 (23.1)
Other	4 (10.0)	3 (7.7)
Unknown	4 (10.0)	9 (23.1)
Weight—kg	58.0 ± 13.9	59.5 ± 14.4
Body mass index—kg/m^2^	22.4 ± 5.0	22.4 ± 5.0
Blood pressure—mmHg		
Systolic blood pressure	142.5 ± 19.3	146.3 ± 14.5
Diastolic blood pressure	65.3 ± 15.0	66.3 ± 14.5
Laboratory		
Hemoglobin—g/dl	11.2 ± 2.7	10.6 ± 0.9
Serum ferritin—ng/ ml	375.1 ± 138.5	368.7 ± 137.9
TSAT—%	28.9 ± 6.8	27.4 ± 8.2
Serum albumin—g/dl	3.5 ± 0.3	3.6 ± 0.2
CRP^b^—mg/L	1.9 [0.9, 4.8]	2.2 [1.0, 5.3]
PTH^b^—pg/ml	427 [221, 552]	270 [151, 457]
SpKt/V	1.9 ± 0.3	1.9 ± 0.3
Medication		
ESA dose^c^—IU/month		
Mean ± SD	33,600 ± 17,513	31,590 ± 13,582
ACEI/ARB—no (%)	13 (32.5)	15 (38.5)
Quality of life		
EQ-5D	12.4 ± 4.3	12.1 ± 5.1

TSAT, transferrin saturation; CRP, c-reactive protein; PTH, parathyroid hormone; SpKt/V, single-pooled Kt/V; ESA, erythropoiesis-stimulating agents; SD, standard deviation; IQR, interquartile range; ACEI, angiotensin-converting-enzyme inhibitor; ARB, angiotensin II receptor blocker.

^a^Data are presented as mean ± standard deviation for continuous variables and number (percent) for categorical variables. Percentages may not total 100 due to rounding.

^b^Non-normal distributed variables are presented as median [interquartile range].

^c^Commercial brands are Eprex, Recormon, Hypercrit, and Hemax.

### Efficacy

The primary outcome, mean monthly ESA dose at month 12, was 35,706 ± 21,637 IU in the 100-mg IV iron group compared with 26,382 ± 14,983 IU in the 200-mg IV iron group (*P* = 0.03) (Fig. 2).

As for secondary outcomes, the death rate was similar throughout (Table 2). Three (3) in the 200-mg IV iron group died from infection. Three (3) in the 100-mg IV iron group died from a cardiovascular event. Twelve (12) patients in the 100-mg IV iron cohort and four in the 200-mg IV iron cohort had absolute IDA. Therefore, 200-mg group had a lower iron rescue therapy (HR 0.30, 95% CI 0.10–0.94, *P* = 0.039) (Fig. 1S). The blood transfusion rate was not significantly different between groups, but higher in the 100-mg IV iron patients. Quality of life, measured by EQ-5D, was similar between groups. Incidence of absolute IDA was significantly higher in the 100-mg IV iron cohort than 200-mg (30% vs. 10.5%, *P* = 0.05).

**Table 2:** Secondary efficacy and safety endpoints at month 12.

Parameter^a^	Iron 100 mg	Iron 200 mg	*P* value
	(n = 40)	(n = 39)	
Secondary efficacy endpoints			
Composite of death, non-fatal myocardial infarction and non-fatal stroke	4 (10.0)	3 (7.7)	0.51
Death from any cause	3 (7.5)	3 (7.7)	1.00
Death from specific causes			0.12
Fatal myocardial infarction	3 (7.5)	0	
Infection	0	3 (7.7)	
Non-fatal myocardial infarction	1 (2.6)	0	1.00
Non-fatal stroke	0	0	NA^b^
Incidence of absolute IDA	12 (30.0)	4 (10.5)	0.049
Blood transfusion	4 (10.0)	1 (2.6)	0.18
Quality of life			
EQ-5D	10.9 ± 4.9	10.2 ± 4.4	0.53
Secondary safety endpoints			
Overall safety events^c^	9 (22.5)	14 (35.9)	0.19
Vascular access thrombosis	1 (2.5)	3 (7.7)	0.29
Hospitalization from any cause	9 (22.5)	13 (33.3)	0.28
Hospitalization from specific causes			0.72
Heart failure	1 (2.5)	2 (5.1)	
Infection	5 (12.5)	6 (15.4)	
Others^d^	3 (7.5)	5 (12.8)	
Iron withholding rate	10 (25.0)	25 (64.1)	< 0.01

ESA, erythropoiesis-stimulating agent; IDA, iron deficiency anemia; NA, not applicable.

^a^Data are presented as mean ± standard deviation for continuous variables and number (percent) for categorical variables. Percentages may not total 100 due to rounding.

^b^If zero cell is presented, *P* value cannot be calculated and is denoted as not applicable.

^c^Overall safety events compose of vascular thrombosis and hospitalization from any cause.

^d^Others are parathyroidectomy and angioplasty.

### Safety

**Figure 3:**
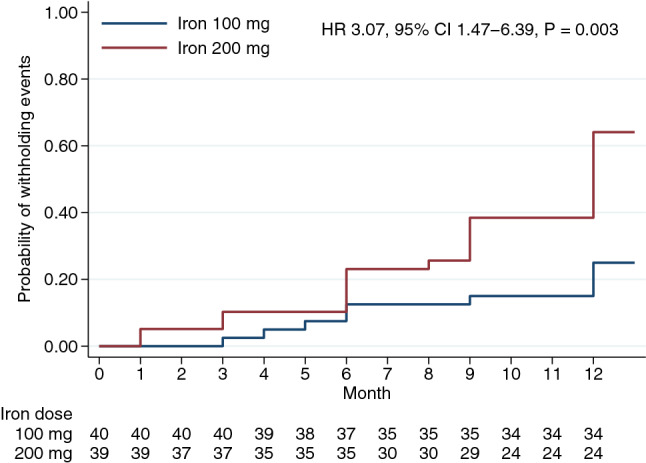
Cumulative incidences of the first withholding intravenous iron events by months.

Overall safety events, including the incidence of vascular thrombosis and hospitalization from any cause, were similar across groups (Table 2). Withholding iron therapy was significantly higher in the 200-mg IV iron regimen than in 100-mg (HR 3.07, 95%CI 1.47–6.39, *P* = 0.003) (Fig. 3).

**Figure 4:**
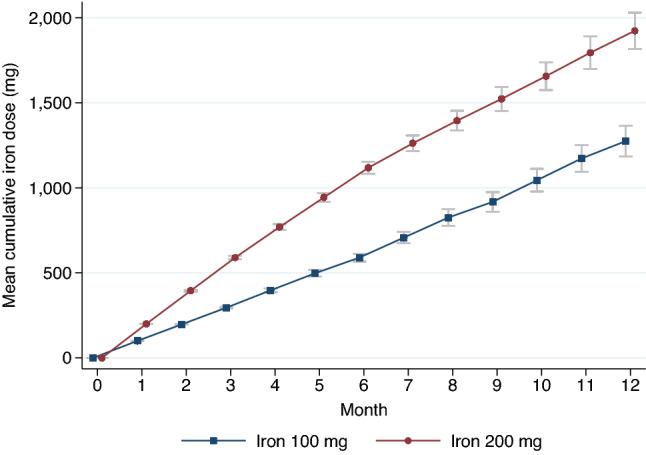
The mean cumulative doses of intravenous iron. The bars indicate 95% confidence intervals. The cumulative doses in each month were compared with the linear random-intercepted model. After month 2, patients in 200-mg intravenous iron group received higher cumulative iron doses than 100-mg group with statistical significance (*P* < 0.05).

**Figure 5:**
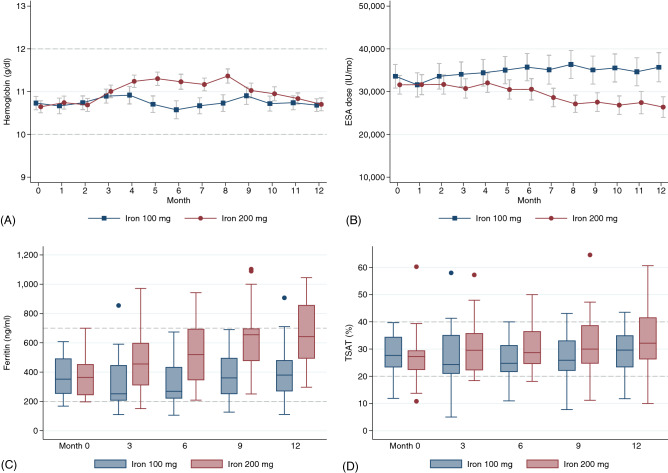
Changes of hemoglobin, ESA doses, ferritin levels, and TSAT by months. (**A**) Hemoglobin change: The target range of hemoglobin level was 10–12 g/dl during the follow-up period (dash bar). The solid bars indicate 95% confidence intervals. (**B**) Monthly erythropoietin dose changes and 95% confidence intervals (solid bars). (**C**) Serial boxplots of ferritin level with the target range of 200–700 ng/ml (dash bar). (**D**) Serial boxplots of transferrin saturation with the target range of 20–40% (dash bar). *Abbreviations:* ESA, erythropoietin; TSAT, transferrin saturation.

Figure 4 shows the mean cumulative doses of IV iron in both groups. The amount of administration was higher significantly in 200-mg IV group after month 2. When comparing laboratory results at month 12 between cohorts, ferritin levels were 413 ± 231 in the 100-mg IV iron group and 668 ± 206 in the 200-mg IV iron group (*P* < 0.001); TSAT was 30.4 ± 10.5% in the 100-mg group and 35.9 ± 14.0% in the 200-mg (*P* = 0.07) (Fig. 5).

Table 3 shows the protocol compliance of IV iron regimens. The patterns between groups were statistically significant (*P* = 0.001). Fifty percent of patients in the 100-mg group and twenty-eight percent in the 200-mg group remained within the protocol. Interestingly, some patients experienced both iron withholding and iron rescue events. Two patients in 100-mg group firstly got IDA (in month 4 and 7, respectively). After 1 g of iron repletion, the iron studies reached above the safety margin; thus, iron withholding occurred. On the contrary, one patient in the 200-mg group had excessive iron supplements at month 7, and iron withholding had continued to month 12. Unfortunately, the laboratory showed a TSAT of 10%, and this patient was given iron rescue therapy after that.

**Table 3:** Protocol compliance of intravenous iron regimens.

Pattern	Iron 100 mg	Iron 200 mg	*P* value^a^
	(n = 40)	(n = 39)	
Following the protocol	20 (50.0)	11 (28.2)	0.001
Iron withholding^b^	8 (20.0)	24 (61.5)	
Iron rescue^c^	10 (25.0)	3 (7.7)	
Both iron withholding and iron rescue^d^	2 (5.0)	1 (2.6)	

Data are presented as number (percent). Percentages may not total 100 due to rounding.

TSAT, transferrin saturation.

^a^Exact probability test was used to calculate the *P* value.

^b^Iron withholding if ferritin > 700 ng/dl or TSAT > 40%.

^c^Iron rescue (total dose of 1 g) in patients with iron deficiency (ferritin < 200 ng/dl or TSAT < 20%).

^d^Iron withholding and iron rescue events occurred in the same patient.

### Subgroup analyses

Post-hoc subgroup analyses of the first incidence of withholding IV iron protocols were stratified by median values of baseline Hb, ferritin, and TSAT (Table 4). Baseline ferritin demonstrated an effect modification, in which a cutoff ≥ 364 ng/dl increased the withholding rate (*P* interaction = 0.04). We cannot find the difference in ESA doses at month 12 between subgroups (Table 1S).

**Table 4:** Subgroup analyses by baseline hemoglobin, ferritin, and transferrin saturation to determine the first withholding intravenous iron events between 100-mg and 200-mg intravenous iron groups.

Subgroup	Iron 100 versus 200 mg		*P* value	*P* value (interaction)
	HR	95% CI		
Overall	3.07	1.47–6.39	0.003	
Baseline hemoglobin^a^—g/dl				
< 10.7	5.22	1.51–18.06	0.009	0.28
≥ 10.7	2.12	0.81–5.58	0.13	
Baseline ferritin^a^—ng/dl				
< 364	1.44	0.55–3.79	0.46	0.04
≥ 364	7.84	2.17–26.24	0.002	
Baseline TSAT^a^—%				
< 27.4	2.43	0.86–6.89	0.10	0.46
≥ 27.4	3.89	1.38–10.98	0.01	

Cox proportional hazard model is used in all subgroup analyses.

TSAT, transferrin saturation; HR, hazard ratio.

^a^Cutoff point of each parameter is selected by median values.

## Discussion

Our study shows that maintenance IV iron at higher doses (200 mg vs. 100 mg) was more effective in reducing ESA doses at month 12. However, the incidence of absolute IDA was significantly higher in the 100-mg IV iron group. Nevertheless, iron therapy was withheld significantly more often in the 200-mg IV iron group. There were more adverse effects in access thrombosis, hospitalization, and infection in the 200-mg IV iron group, without statistical significance.

Maintenance IV iron in incident hemodialysis patients has been previously studied. As mentioned, the PIVOTAL trial^8^ tried to compare a proactive high-dose iron regimen (400 mg of iron sucrose per month unless serum ferritin > 700 ug/dl or a TSAT ≥ 40%) with a reactive low-dose regimen (0–400 mg of iron sucrose as required to achieve minimum ferritin of 200 ug/dl and a TSAT of 20%). The proactive group had a lower primary endpoint event, defined as the composite of nonfatal myocardial infarction, nonfatal stroke, hospitalization for heart failure, or death from any cause, of HR 0.85 (95% CI, 0.73–1.00). This finding demonstrated that a maintenance supplement was superior to a reactive one. Nonetheless, the prescribed study iron dose of 400 mg iron sucrose in the proactive group could not actually be administered due to safety cutoff limits: ferritin level of 700 µg/dl or a TSAT of 40%. The median monthly iron was 264 mg; thus, it remained challenging to determine appropriate doses of maintenance IV iron therapy.

Susantitaphong et al.^11^ conducted a randomized control trial in chronic hemodialysis patients with Hb 8–12 g/dl, ferritin 200–400 ng/ml, and TSAT < 30%. They prescribed IV iron as a maintenance regimen to maintain either a high ferritin level (600–700 µg/dl) or a low one (200–400 µg/dl). ESA doses were adjusted to keep Hb levels at 10–12 g/dL. After six months of follow-up, they found a significant decrease in the erythropoietin resistance index in the high ferritin group compared to the low ferritin one. The average IV iron dose in their study was 190 mg per month in the high ferritin group and 110 mg per month in the low one. This iron dose was close to what we used in our study. Susantitaphong et al. concluded that high ferritin could decrease ESA-dose requirements after 6 months of maintenance iron therapy. However, our study did not stratify our groups into high or low ferritin and instead focused on fixed doses of iron therapy.

Theoretically, the appropriate dose of iron supplementation should be equivalent to iron loss. In hemodialysis patients, losing residual blood in the circuit and chronic blood loss in the gastrointestinal tract are the significant causes of iron loss, approximately 1–3 g per year and equivalent to 83–250 mg per month^6,7^. The Dialysis Outcomes and Practice Patterns Study (DOPPS)^12^, a multinational prospective cohort study, suggested typical maintenance iron dosing of 100–200 mg per month effectively maintained Hb levels and kept ferritin levels TSAT stable. The limitations of DOPPS were a short-term follow-up (3-month average) and the nature of an observational study, which may have created residual confounding and causal interpretation of treatment effects. Mortality and hospitalization were increased in patients receiving IV iron sucrose ≥ 300 mg per month versus the dose of 100–199 mg^13^.

Another observational study, using 58,058 hemodialysis patients from DaVita dialysis clinics, showed that IV iron > 400 mg per month was associated with higher all-cause and cardiovascular mortality rates^14^. As mentioned, our study chose monthly doses of 100-mg and 200-mg iron sucrose, close to the iron loss range mentioned above and supported by DOPPS^12^. Our results showed that 200 mg per month was more effective in reducing ESA doses at 12 months; however, the iron withholding rate was significantly higher in this group (64.1%). This implies that 200 mg of IV iron per month may be too much for long-term maintenance therapy.

Safety is always an essential consideration in prescribing iron. IV iron may increase oxidative stress and lead to cardiovascular and infectious complications^1,5,15^. Like the PIVOTAL trial^8^, our study set a ceiling iron level of > 700 ng/dl of serum ferritin levels or > 40% of TSAT. The PIVOTAL trial did not show an increase in hospitalization, infection rate, and vascular access thrombosis in the higher group^16^. Still, it did observe higher infection events and hospitalization in patients who were hemodialysed via a catheter^17^. Our study found slightly higher access thrombosis, and hospitalization but no statistical significance. For the current knowledge, these targets of serum ferritin and TSAT have been proven for infectious safety in clinical trials. Nevertheless, there are some conflicting results from observational studies on whether the IV iron dose is associated with the incidence of infection^18,19^.

Additionally, the withholding rates in our study were up to 25% and 64% in 100-mg and 200-mg IV iron. After performing subgroup analysis, baseline ferritin ≥ 364 ng/dl was significantly associated with withholding event. Therefore, in patients with high baseline ferritin levels, we suggest trying 100-mg IV iron first and promptly increasing the dose if the ferritin level does not increase appropriately. This may be less risky than starting with a fixed dose of 200 mg.

To our knowledge, this is the first clinical trial of fixed-dose protocol maintenance IV iron. The strength of our study was a randomized, double-blinded, controlled trial, which minimized confounding and other bias relatively well. Secondly, our iron protocol was simple and easy to comply with. However, there were many limitations. First, this study was conducted at a single center, affecting generalizability. Second, the study population was relatively small, and our follow-up period was limited to 12 months. We cannot conclude the impact of the protocol on cardiovascular outcomes and critical adverse events, such as thrombosis and infection. Third, the actual monthly doses (174 and 116 mg) were different from the protocol because of the high withholding rate in the 200-mg group and the high rate of IDA in the 100-mg group, representing that fixed-dose is not fit for all patients. Fourth, clinicians could recognize when rescue iron therapy or withholding events occurred so that ESA dose adjustment may be affected.

A maintenance IV iron regimen of 200 mg per month was more effective than the 100-mg dose to minimize ESA doses in hemodialysis patients. Nonetheless, the iron withholding rate was higher. The stepping-up protocol may be an appropriate option in patients with high baseline ferritin if within safety parameters.

## Supplementary Information

Supplementary Information.

## Data Availability

The datasets generated during and/or analyzed during the current study are available from the corresponding author upon reasonable request.
