# Supplementary material for: Maintenance intravenous iron in hemodialysis patients to minimize erythropoietin doses: a double-blinded, randomized controlled trial (the MAINTAIN IRON trial)
Source: Sci Rep. 2023 Jan 23;13:1287. doi: 10.1038/s41598-023-28440-3 (PMC9870895; doi:10.1038/s41598-023-28440-3)
Supplement: Supplementary file 1 — Supplementary Information. [file 41598_2023_28440_MOESM1_ESM.docx]

**
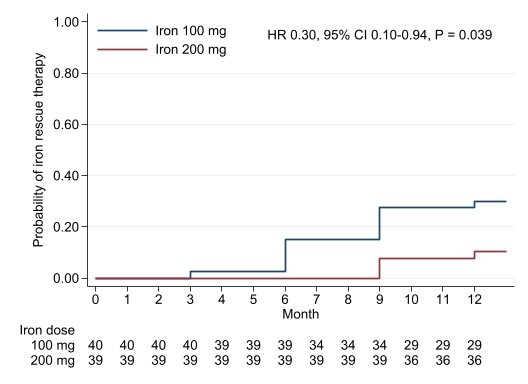
**

**Figure 1S.** Cumulative incidences of the intravenous iron rescue therapy by months.

| **Subgroup** | **Iron 100 vs 200 mg** | | ***P* value** | ***P* value (interaction)** | |
| --- | --- | --- | --- | --- | --- |
|  | **Difference^a^** | **95% CI** |  |  |  |
| Overall | -5,676 | -12,753 – 1,401 | 0.11 |  |  |
| Comorbidity |  |  |  |  |  |
| Diabetes | -4,782 | -16,118 – 6,554 | 0.40 | 0.73 |  |
| Non-diabetes | -7,312 | -16,616 – 1,991 | 0.12 |  |  |
| Vascular access type |  |  |  |  |  |
| Permanent catheter | -6,333 | -16,334 – 3,667 | 0.20 | 0.99 |  |
| AVF or AVG | -6,320 | -15,563 – 2,924 | 0.18 |  |  |
| ^a^Difference represents a mean difference of monthly unit of erythropoietin at month 12  Linear regression model is used in all subgroup analyses.  Abbreviations: AVF, arteriovenous fistula; AVG, arteriovenous graft. | | | | |  |

**Table 1S.** Subgroup analyses by diabetes mellitus and vascular access type ferritin to determine the difference of erythropoietin at month 12 between 100-mg and 200-mg intravenous iron groups.
